# Emilia-Romagna Surgical Colorectal Cancer Audit (ESCA): a value-based healthcare retro-prospective study to measure and improve the quality of surgical care in colorectal cancer

**DOI:** 10.1007/s00384-022-04203-w

**Published:** 2022-07-02

**Authors:** Ilaria Massa, Federico Ghignone, Giampaolo Ugolini, Giorgio Ercolani, Isacco Montroni, Patrizio Capelli, Gianluca Garulli, Fausto Catena, Andrea Lucchi, Luca Ansaloni, Nicola Gentili, Valentina Danesi, Maria Teresa Montella, Mattia Altini, The ESCA Collaborative Group, William Balzi, Andrea Roncadori, Giacomo Ferri, Simona Gallo, Giuseppa Di Genova, Nicola Albertini, Davide Zattoni, Stefano Bolzon, Andrea Avanzolini, Davide Cavaliere, Daniela Di Pietrantonio, Leonardo Solaini, Leonardo Luca Chiarella, Giovanni Taffurelli, Federico Mazzotti, Giacomo Frascaroli, Francesco Pasini, Francesca Di Candido, Filippo Banchini, Andrea Romboli, Gerardo Palmieri, Luigi Conti, Enrico Luzietti, Mattia Portinari, Basilio Pirrera, Enrico Fantini, Monari Francesco, Gianmarco Palini, Giacomo Stacchini, Alessandra Sguera, Erika Picariello, Enrico Faccani, Chiara Gurioli, Giulia Vitali, Michele Grassia, Laura Agostinelli, Luigi Romeo, Gianluca Senatore

**Affiliations:** 1 Outcome Research, Healthcare Administration, IRCCS Istituto Romagnolo per lo studio dei tumori (IRST) “ Dino Amadori”, Meldola, Italy; 2 U.O. Chirurgia Generale, Hospital “Santa Maria delle Croci”, AUSL, Ravenna, Romagna Italy; 3 U.O. Chirurgia Generale e Terapie Oncologiche avanzate, Hospital “GB. Morgagni-L.Pierantoni”, AUSL, Forli, Romagna Italy; 4 U.O Chirurgia Generale, Hospital “degli Infermi”, AUSL, Faenza, Romagna Italy; 5 Department of Surgery, Hospital “G. Da Saliceto”, Piacenza, AUSL, Piacenza, Italy; 6 U.O. Chirurgia Generale, Hospital “Infermi”, AUSL, Rimini, Romagna Italy; 7 General, Emergency and Trauma Surgery Dept., Bufalini Hospital, AUSL, Cesena, Romagna Italy; 8 U.O. Chirurgia Generale, Hospital “Ceccarini”, AUSL, Riccione, Romagna Italy; 9 Department of Surgery, Fondazione IRCCS Policlinico San Matteo, University of Pavia, Pavia, Italy; 10 Healthcare Administration, AUSL of Romagna, Ravenna, Italy

**Keywords:** Audit, Feedback, Benchmarking, Quality improvement, Colorectal cancer, Surgery

## Abstract

**Purpose:**

Surgery is the main treatment for non-metastatic colorectal cancer. Despite huge improvements in perioperative care, colorectal surgery is still associated with a significant burden of postoperative complications and ultimately costs for healthcare organizations. Systematic clinical auditing activity has already proven to be effective in measuring and improving clinical outcomes, and for this reason, we decided to evaluate its impact in a large area of northern Italy.

**Methods:**

The Emilia-Romagna Surgical Colorectal Audit (ESCA) is an observational, multicentric, retro-prospective study, carried out by 7 hospitals located in the Emilia-Romagna region. All consecutive patients undergoing surgery for colorectal cancer during a 54-month study period will be enrolled. Data regarding baseline conditions, preoperative diagnostic work-up, surgery and postoperative course will be collected in a dedicated case report form. Primary outcomes regard postoperative complications and mortality. Secondary outcomes include each center’s adherence to the auditing (enrolment rate) and evaluation of the systematic feedback activity on key performance indicators for the entire perioperative process.

**Conclusion:**

This protocol describes the methodology of the Emilia-Romagna Surgical Colorectal Audit. The study will provide real-world clinical data essential for benchmarking and feedback activity, to positively impact outcomes and ultimately to improve the entire healthcare process of patients undergoing colorectal cancer surgery.

**Clinical trial registration:**

The study ESCA is registered on the clinicaltrials.gov platform (Identifier: NCT03982641).

**Supplementary Information:**

The online version contains supplementary material available at 10.1007/s00384-022-04203-w.

## Introduction

*“If you can not measure it, you can not improve it” (Lord Kelvin)*

**Fig. 1:**
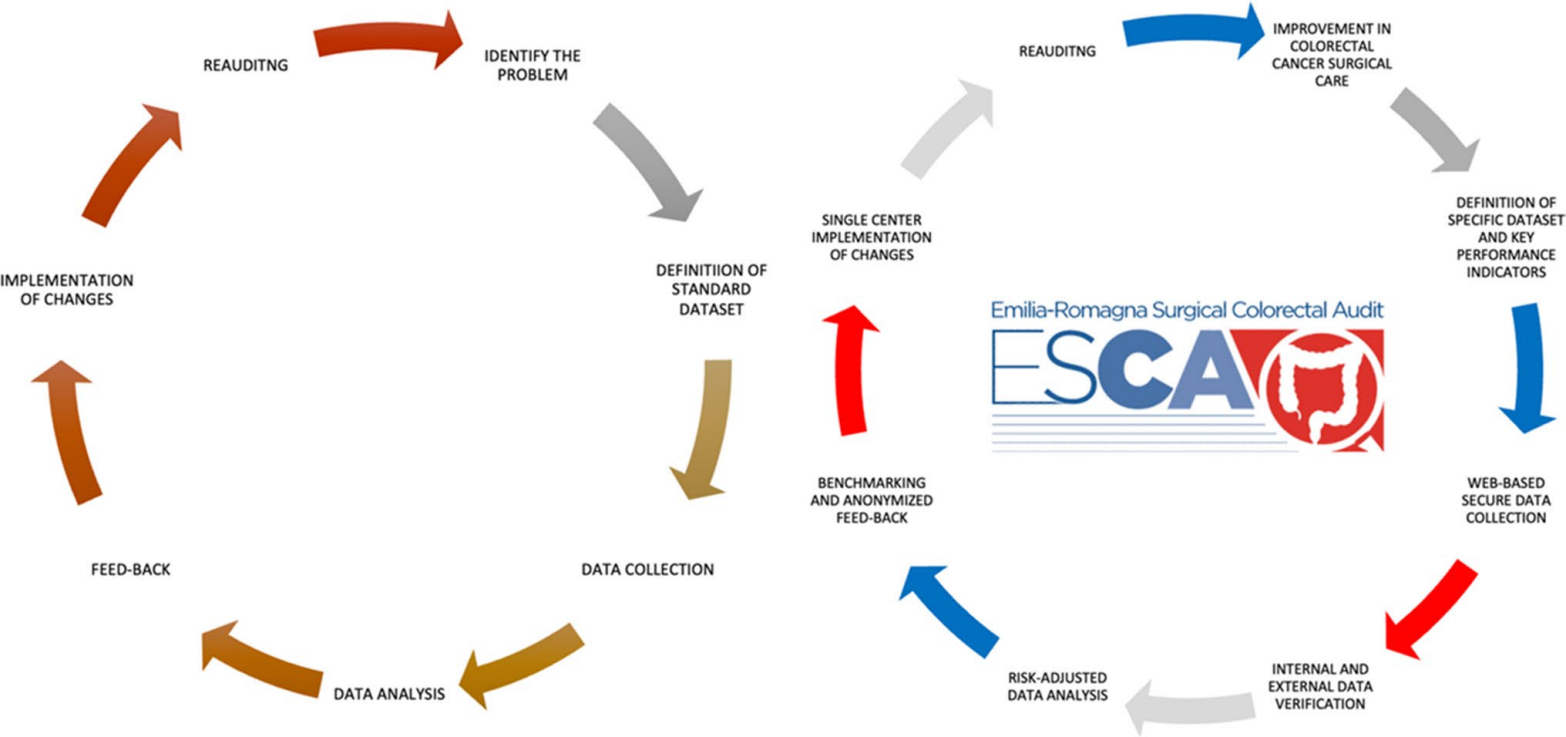
General and ESCA clinical auditing process

A clinical audit is a process which assesses whether healthcare is meeting standards, with the capacity to reduce care disparities as well as being a method to cut unnecessary costs. Where indicated, changes are implemented at an individual, team, or service level, and further monitoring is used to confirm improvement in healthcare delivery [1]. Reliable data, meticulous measurement, and systematic feedback to participants about their performances are the three pillars at the base of an effective clinical audit. Only good data can bring understanding of what does and does not improve the quality of healthcare, and a continuous assessment process allows for concrete advancement (Fig. 1).

The so-called “Hawthorne effect” (the awareness of being monitored positively influences the behavior of monitored individuals) also helps improve the process, as already widely demonstrated [2–6].

Medical literature offers a vast number of international clinical audits focused on colorectal cancer surgery [7–10]. The Dutch Colorectal Audit (DCRA) probably represents one of the most effective initiatives aiming at improving surgical quality outcomes [7]. DCRA started in 2009 and after just 8 years of activity more than 70,000 patients were included, showing a dramatic decrease in postoperative mortality from 3.4 to 1.8% for colon cancer and from 2.3 to 1% for rectal cancer [10]. Moreover, a significant reduction in costs for the entire healthcare system was observed along with a reduction in complications [11] and an optimization of resources such as the reduction in preoperative radiation therapy for rectal cancer without any impact on oncological outcomes[12].

In 2020, more than 43,000 people in Italy were diagnosed with colorectal cancer (CRC). Of these, approximately 800 of them lived in proximity of the centers taking part in this study [13]. Looking at the administrative data of the Emilia-Romagna region, huge variability was also observed in terms of volume and surgical outcomes, ranging respectively from 36 to 290 procedures per hospital and 2.26% up to 9.45% for 30-day mortality [14].

Following the path of the DCRA experience and given the heterogeneity of surgical outcomes among the hospitals in our region, we decided for the first time in Italy to promote a systematic clinical auditing pilot study focused on CRC surgical care and its possible benefit on outcomes.

## Methods

### Data governance and ethics

The protocol has been described according to the Standard Protocol Items: Recommendation for the Investigational Trials (SPIRIT) checklist [15] (Appendix). The ethical committee approved the project for each of the centers taking part according to local regulations, and it has been registered on the clinicaltrials.gov platform (Identifier: NCT03982641). The Romagna Ethical Committee (CEROM) approval number is 2278.

### Study organization, administration, and governance

The Emilia-Romagna Surgical Colorectal Audit (ESCA) is a multicenter, retro-prospective, observational non-profit study promoted by the IRCCS Istituto Romagnolo per lo Studio dei Tumori (IRST) “Dino Amadori” in Meldola and the Local Health Agency of Romagna (AUSL Romagna).

Members of the surgical units participating in the study contributed to its design gave feedback and reviewed the study protocol. ESCA is overseen by a study steering committee composed of surgeons, epidemiologists, statisticians, IT specialists, and healthcare administrators.

ESCA received a financial support from Johnson & Johnson Medical S.p.a Ethicon Division for data management and monitoring.

The objectives of the study are as follows:

1. To systematically collect data of all patients undergoing surgical treatment for primary CRC from diagnosis up to 6 months after surgery;
2. To evaluate risk adjusted post-operative complications and mortality;
3. To evaluate adherence to the clinical audit activity of each participating center;
4. To evaluate the possible positive effects on outcomes, both clinical and economic, of systematic measurement and monitoring (Hawthorne effect).

### Setting and study population

Patients will be enrolled from the 7 hospitals located in the Emilia-Romagna region in Northern Italy (Table 1), during a 54-month study period. The recruitment target is around 800 patients per year on the basis of demographic data reported in previous years.

**Table 1:** Participating centers

Participating centers
Hospital “Degli Infermi” — Faenza
Hospital “S.Maria delle Croci” — Ravenna
Hospital “Morgagni-Pierantoni” — Forlì
Hospital “Infermi” — Rimini
Hospital “Bufalini” — Cesena
Hospital “Ceccarini” — Riccione
Hospital “Giovanni da Saliceto” — Piacenza

### Inclusion criteria

All consecutive patients undergoing surgery for primary CRC between 15 April 2019 and 31 December 2023 will be enrolled in the study. All types of surgical procedures are included, irrespective of setting (urgent/emergent or elective), intent (curative or palliative), and approach (open or minimally invasive). Eligible patients are asked to sign an informed consent form. Cognitive impairment is not considered an exclusion criterion if informed consent is obtained by an appropriate healthcare proxy.

### Exclusion criteria

Patients with multiple synchronous primary tumors are excluded from the analysis. Patients who are unwilling to sign an informed consent form are also excluded.

### Patient’s withdrawal

Participating subjects have the right to withdraw at any time for any reason; data will be collected until the patient’s withdrawal point.

### Study outcomes

The primary objective of this analysis is to assess the frequency of post-operative complications, unplanned re-interventions, re-admissions, and mortality rates (at 30, 90, and 180 days after surgery).

Secondary outcomes include adherence to the clinical audit by each center (intended as the percentage of enrolled eligible patients) along with the study timeframe and possible positive effects on outcomes, both clinical and economic, of systematic measurement and monitoring.

### Key performance indicators, case-mix, benchmarking, and feed-back activity

A core set of key performance indicators (KPIs) will be assessed to measure performance and evaluate the quality of colorectal cancer surgery across the participating centers (Table 2). Each KPI will be estimated both as unadjusted and risk adjusted for differences in patients’ characteristics for a fair comparison among the participating hospitals.

**Table 2:** Overview of the selected key performance indicators

**ID**	**Indicator title**	**Indicator description**	**Indicator type**
1	Multidisciplinary discussion	Rate of patients discussed at a multidisciplinary meeting (MDM) before surgery	Colon and rectal cancer
2	Neoadjuvant treatment	Rate of patients receiving neoadjuvant treatment (radiation therapy, chemo-radiation, chemotherapy, total neoadjuvant treatment)	Rectal cancer
3	Minimally invasive resections	Rate of patients undergoing minimally invasive resection	Colon and rectal cancer
4	Conversion	Conversion rate from minimally invasive to open surgery at any time during the procedure	Colon and rectal cancer
5	Anastomotic leak	Rate of anastomotic leakage	Colon and rectal cancer
6	Anastomosis	Rate of patients undergoing anastomosis after low anterior resection	Rectal cancer
7	Loop ileostomy	Rate of loop ileostomy creation after low anterior resection	Rectal cancer
8	Length of stay (LOS)	Rate of patients discharged within postoperative days 3 and 5	Colon and rectal cancer
9	Postoperative complications	Rate of patients with severe postoperative complications (grade ≥ 3 according to Clavien–Dindo classification)	Colon and rectal cancer
10	Quality of total mesorectal excision (TME)	Rate of patients with TME grade 1 or 2	Rectal cancer
11	Positive circumferential resection margins (CRM)	Rate of reported positive CRMs (less than or equal to 1 mm)	Rectal cancer
12	Lymph-nodes yield	Rate of patients for who received adequate lymphadenectomy (≥ 12 lymph-nodes)	Colon and rectal cancer
13	Unplanned reintervention	Rate of patients with an unplanned return to theatre within 30 days after index surgery	Colon and rectal cancer
14	Unplanned readmission	Rate of patients with unplanned readmission within 30 days after discharge/transfer from surgical unit	Colon and rectal cancer
15	Postoperative mortality	Rate of patients who died 30,90, or 180 days after surgery	Colon and rectal cancer

Every 12 months, an anonymized report on volume and KPIs will be delivered to each hospital comparing data of all the participating centers. A set of relevant case-mix variables (patients’ frailty, tumor burden, type of surgery) will be also identified in order to produce a reliable comparison of outcomes between hospitals and give each center case-mix adjusted outcomes.

Data collected during the first 18 months (from April 2019 to October 2020) will be analyzed in order to evaluate preliminary results on primary and secondary outcomes. The first preliminary analysis will be used as benchmark to further implement the auditing activity.

**Fig. 2:**
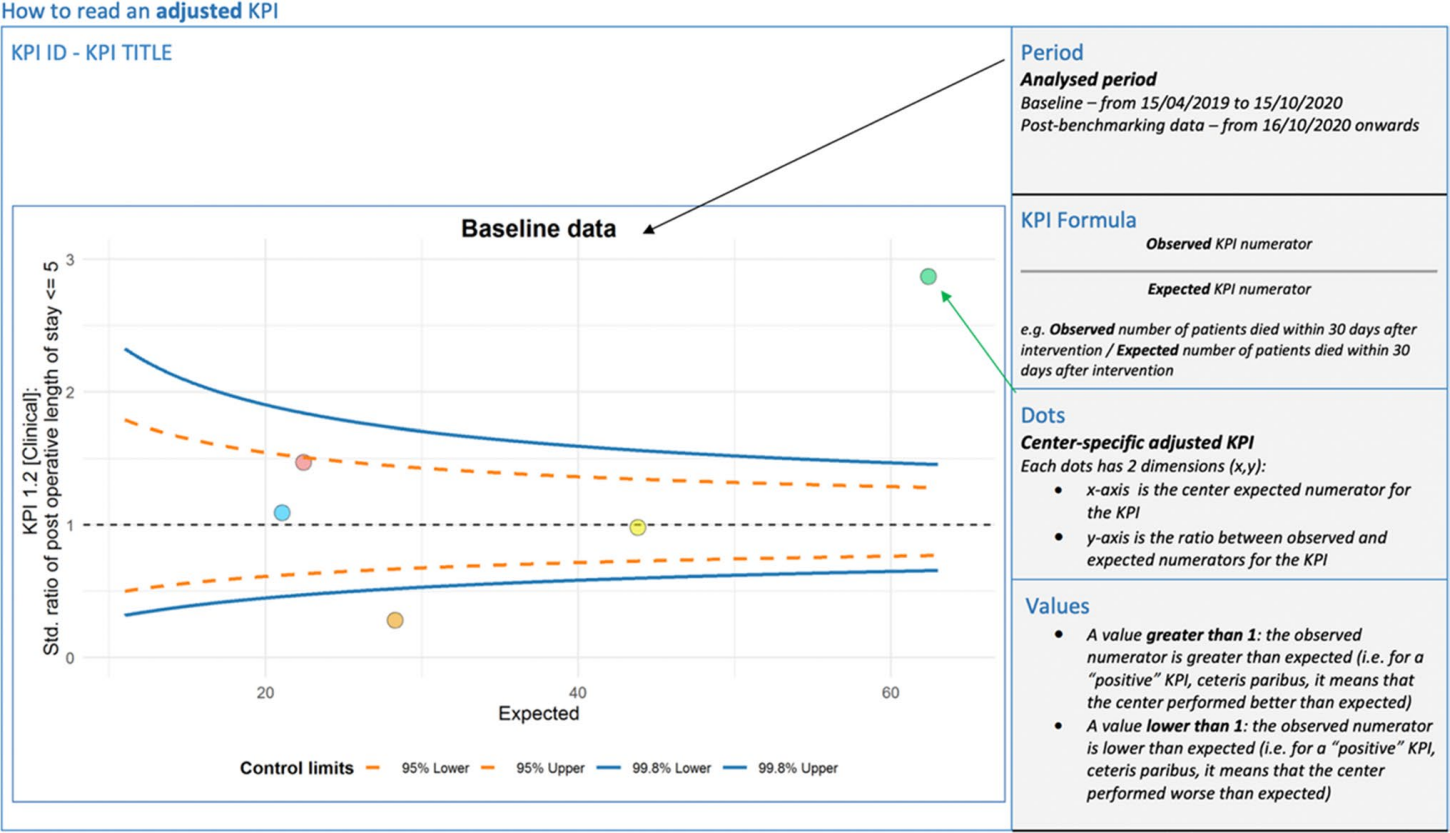
Case-mix adjusted data will be presented using funnel plots with a 95% confidence interval

Case-mix adjusted data will be presented using funnel plots with a 95% confidence interval (Fig. 2).

Reports of the enrolment rate, obtained by comparing the number of enrolled patients to the hospital discharge cards (HDC), will be sent to each center every 3 months. The aim of this feedback activity is to progressively obtain a 100% enrolment of eligible patients and thus provide “real-world data” that avoid bias related to patient selection.

### Data collection and quality control

The data collection and management for this paper are performed using the OpenClinica open-source software for Electronic Data Capture (EDC), version 3.1 (Copyright OpenClinica LLC and collaborators, Waltham, MA, USA, www.OpenClinica.com). Case report forms are filled in for each patient by trained healthcare providers (attendings, residents) or properly trained data managers. Operative and postoperative data are retrieved both from electronic medical charts and administrative databases. Surgical data analysis, including detection of postoperative complications, will be done under the supervision of a staff surgeon.

Quality control and data authenticity will be performed by data managers and clinical research coordinators. Clinical data will be compared to routinely collect administrative information retrieved from regional registries to ensure reliability and completeness and to avoid selection bias. Registration of each patient is automatically linked to the regional administration database, which by law receives notification on all patients deceased in Emilia-Romagna.

### Study variables

The full data set is composed of up to 172 possible variables, and it is organized in sections as follows.

#### Preoperative assessment

Preoperative functional assessment is conducted using the Eastern Collaborative Oncology Group Performance Status (ECOG PS) [16] and the American Society of Anaesthesiology (ASA) score [17, 18]. Patients aged ≥ 70 years are screened for frailty with the Katz Activities of Daily Living (ADL) [19] and the Flemish version of the Triage Risk Screening Tool (fTRST) [20, 21]. Comorbidities are assessed using the age adjusted Charlson Comorbidity Index (CCI) [22, 23] and the presence of malnourishment is evaluated using the nutritional risk score (NRS) [24]. All screening tests are reported in detail in Table 3. Baseline evaluation further includes information on the living conditions before surgery and polypharmacy.

**Table 3:** Preoperative functional status assessment tools

Test	Range
American Society of Anaesthesiologists (ASA) score	1–5
Eastern Collaborative Oncology Group Performance Status (ECOG-PS)	0–5
Katz Activities of Daily Living (ADL)	0–6
Flemish version of the Triage Risk Screening Tool (fTRST)	0–6
Nutritional risk screening (NRS)	0–3
Age-adjusted Charlson Comorbidity Index (CCI)	0–38

The diagnostic work-up is then assessed including the following items:

1. Number and location of primary cancer;
2. Presence/absence of distant metastases;
3. Date of preoperative endoscopy and pathology report of biopsy;
4. Presence of cancer-related preoperative complications (anemia, colonic obstruction, perforation);
5. Date and type of preoperative imaging studies (CT scan, MRI, PET-CT) as appropriate.

For rectal cancer patients undergoing pelvic MRI and/or neoadjuvant treatments, specific items will be also collected as reported in Table 4.

**Table 4:** Pelvic MRI features for rectal cancer and type of neoadjuvant treatment

PREOPERATIVE PELVIC MRI	POSSIBLE ANSWERS
Mucinous features	yes/no
Distance from anal verge	value [mm]
Distance from pubo-rectal sling	value [mm]
Cranio-caudal extension	value [mm]
Presence of extra-mural vascular invasion (EMVI)	yes/no
Invasive margin site assessed?	yes/no
Invasive margin site location	Anterior Posterior Right lateral Left lateral Circumferential
Mesorectal extra-mural invasion and depth	yes/no [mm]
Lymph node status	N0/N+
Minimal distance from mesorectal fascia	value [mm]
Location of minimal distance from mesorectal fascia	Anterior Posterior Right lateral Left lateral
mrTNM	[User should report the tumour node metastasis staging]

TYPE OF NEOADJUVANT TREATMENT	
Short course radiation therapy (5×5)	yes/no
Standard long course chemo-radiotherapy	yes/no
Total neoadjuvant therapy (TNT)	yes/no
Chemotherapy alone	yes/no

RESTAGING PELVIC MRI	
Mucinous features	yes/no
Distance from anal verge	value [mm]
Distance from puborectal sling	value [mm]
Cranio-caudal extension	value [mm]
Presence of EMVI	yes/no
Invasive margin site assessed?	yes/no
Invasive margin site location	Anterior Posterior Right lateral Left lateral Circumferential
Mesorectal extra-mural invasion and depth	yes/no [mm]
Location of minimal distance from mesorectal fascia	Anterior Posterior Right lateral Left lateral
Lymph node status	N0/N+/na
Minimal distance from mesorectal fascia	value [mm]
Location of minimal distance from mesorectal fascia	Anterior Posterior Right lateral Left lateral
ymrTNM	[User should report the tumour-node-metastasis staging after neoadjuvant treatment]

#### Surgery

Type of surgical procedure, regimen, length of surgery, and operative technique is collected. Possible intraoperative complications are identified and specified as well as the need for intraoperative blood transfusions. The radicality of surgery is assessed as well as type of anastomosis including characteristics and technique. If stoma is created, details are reported as appropriate. All surgical items are reported in detail in Table 5.

**Table 5:** Surgical variables

SURGERY	POSSIBLE ANSWERS
Colonic stent preoperatively placed?	yes/no
Surgical regimen	Elective Urgent Unknown
Surgical intent	Curative Palliative Unknown
Surgical technique	Laparotomy Laparoscopy Robotic TEM Transanal open TAMIS taTME Unknown
Conversion	yes/no
Time to conversion	Early (< 60 minutes) Late (> 60 minutes)
Reason of conversion	Reactive Strategic
Surgical procedure	Ileo-cecal resection Right colectomy Extended right colectomy Transverse colon resection Splenic flexure resection Left colectomy Low anterior resection with PME Sigmoid resection Subtotal colectomy Abdomino-perineal excision Total proctocolectomy Transanal local excision Explorative procedure
Type of abdomino-perineal resection:	Standard APR (APE) Extralevator APR (ELAPE) Pelvic exenteration Beyond TME (APR with sacrectomy)
Type of pelvic exenteration: (specify organ included into the resection)	Cystectomy Prostatectomy Istero-annessectomy
Intra-operative complications	yes/no
Type of Intra-operative complication	Splenic injury Biliary injury Small bowel injury Vaginal injury Urological injury Peritoneal perforation during transanal procedure
Additional resections	Ileal resection Minor hepatic resection Major hepatic resection Other abdominal/pelvic organs Peritonectomy (including HIPEC) Lung resection Other (specify)
Intra-operative blood transfusion?	yes/no
Length of surgery	value [minutes]
Radicality	R0/R1/R2
Anastomosis	yes/no
Type of anastomosis—1	Hand-sewn Stapled
Type of anastomosis—2	Ileo-colonic Colo-colonic Colo-rectal Ileo-rectal Colo-anal Ileo-anal
Type of anastomosis—3	Intra-corporeal Extra-corporeal Not applicable
Stoma?	yes/no
Type of stoma	End ileostomy End colostomy Loop ileostomy Loop colostomy

#### Postoperative course

Need of the post-operative intensive care unit (ICU), length of ICU stay, postoperative LOS, and discharge settings are all registered. Thirty-day postoperative morbidity is collected and classified according to Clavien–Dindo classification [25, 26]. Cumulative burden of postoperative complications is calculated for each patient according to the comprehensive complication index [27]. If re-intervention is needed, the reason for reintervention, operative procedure, approach, and need for postoperative ICU stay are all collected (Table 6).

**Table 6:** Postoperative course

POSTOPERATIVE COURSE	POSSIBLE ANSWERS
ICU stay	yes/no
Length of ICU stay	value [days]
Postoperative length of stay	value [days]
Discharge/transfer setting	Other ward Rehabilitation Home Nursing home
Complications according to Clavien–Dindo	yes/no (if yes user should report type of complication and grade)
Anastomotic leak?	yes/no
Bleeding requiring transfusion?	yes/no
ICU transfer because of postoperative complications?	yes/no
Re-intervention within 30 days?	yes/no
Re-intervention reason	[specify]
Re-intervention: procedure	[specify]
Pre-reintervention setting	Surgical ward Other ward ICU Home
Post-reintervention ICU stay	yes/no

#### Pathology

The pathology report includes cancer type and grade of differentiation according to the WHO classification [28], the number of retrieved and positive lymph-nodes, and lympho-vascular and perineural invasion. For patients with rectal cancer, additional information includes extra mural vascular invasion (EMVI), evaluation of distal and circumferential margins, TME quality according to the Quirke classification [29, 30], and grade of regression following neoadjuvant treatment according to Ryan/CAP [31]. Final tumor stage is reported according to the 7th edition of the TNM cancer staging system [32] (Table 7).

**Table 7:** Pathology and postoperative oncological treatment

PATHOLOGY	POSSIBLE ANSWERS
Histological type	[User should report histologic type of tumour]
Number of lymph-nodes retrieved	value
Number of metastatic lymph-nodes	value
Grade of differentiation	Low/high
Lymphatic invasion	yes/no
Vascular invasion	yes/no
Perineural invasion	yes/no
pTNM	[User should report pathologic tumour-node-metastasis staging]
Pathology — rectal cancer	
EMVI	
Free distal margin	yes/no
Free radial margin	yes/no
TME quality according to Quirke	Grade 1 Grade 2 Grade 1 Not assessed
Grade of regression after neoadjuvant therapy	Grade 0 Grade 1 Grade 2 Grade 3 Not assessed
pTNM	[User should report pathologic tumour-node-metastasis staging]
Postoperative chemotherapy	yes/no
Postoperative radiotherapy	yes/no

#### Follow-up

After hospital discharge, any postoperative oncological treatment will be reported (adjuvant chemotherapy and/or radiation therapy). Possible changes in living conditions, considered as a proxy for functional recovery, will be reported. Any emergency department access until postoperative day 30 and the reason for it will be collected as well as the need for re-hospitalization. Mortality at 30, 90, and 180 days postoperatively will be collected, together with the cause of death (Table 8).

**Table 8:** Follow up

30 DAYS - FOLLOW UP	POSSIBLE ANSWERS
Emergency room access because of surgical complications	yes/no
Re-admission from first surgery	yes/no
Reason for readmission	surgical complication complication not related to surgery other
In-hospital death	yes/no
Cause of death	Disease progression Complications other medical issues unknown
Patient status at 30 days	alive/death

90 DAYS – FOLLOW UP	
Patient status at 30 days	alive/death

180 DAYS – FOLLOW UP	
Patient status at 30 days	alive/death

## Statistical considerations

All the analyses will be performed considering tumor location (colon or rectum) as a stratification factor. For continuous variables, the arithmetic mean and standard deviation (SD), as well as the median value and minimum–maximum, will be presented. Absolute frequencies together with the percentage relative frequencies will be reported while summarizing qualitative variables.

To evaluate the performance of surgical activity, absolute numbers and relative percentages of each performance indicator reported will be calculated. The mortality rate will be defined as the number of patients who died within 30, 90, and 180 days after surgery. Graphical representation will also be used: funnel plots will be displayed for each KPI, both in unadjusted and adjusted versions.

Appropriate statistical models (i.e., mixed models, logistical regression, multilevel models) will be developed to evaluate the relationship among analyzed indicators and potential explanatory factors as well as for standardization/adjustment purposes. Main covariates will include age, gender, ASA score, CCI, surgery setting (urgent vs. elective), and ECOG.

Hospitals will be used as random effects to account for the presence of possible variability among hospitals. Other exploratory subgroup analyses will be performed.

Furthermore, to assess the effects of continuous monitoring and benchmarking on clinical outcomes, a before-and-after approach will be used. Specifically, assuming the time from the first subject surgery until the release of the first benchmarking report as the reference period (baseline, i.e., “before” period), the analysis will evaluate any significant changes occurred afterwards. Any changes on the outcomes will be, therefore, attributed to the benchmarking effect.

Missing values will be replaced and estimated using multiple imputations. A two-sided 95% confidence interval (95% CI) will be reported as appropriate.

Statistical analysis will be performed using R statistical software (v. 4.0.6) — www.r-project.org.

## Discussion

Surgery is the main treatment modality for stages I–III colorectal cancer and frequently represents the most effective choice even in a palliative setting. Despite massive improvements in perioperative care and techniques, colorectal cancer surgery is still associated with a significant burden of postoperative complications which result in greater healthcare costs and severe functional sequalae for patients. If the value of healthcare is maximizing quality care at minimal cost [33], the large-scale participation in an audit, which constantly measure the care quality and the resources used associated for a benchmark feedback, represents a unique opportunity to significantly improve healthcare and limit expenditure.

Administrative data have already revealed their limitations when used to evaluate quality of care and can lead to misinterpretation when used to measure composite postoperative outcomes of complex and/or frail patients [34, 35]. However, together with mortality registries, they are essential for quality check control (enrollment rate) and completeness of data entry for specific items (re-admission rate, emergency department admission after discharge): for this reason, they were integrated into the entire ESCA auditing process.

Clinical data are more difficult and expensive to collect than administrative data, but these challenges are far outweighed by the opportunity clinical data can create in obtaining reliable information on the entire clinical process, ultimately improving quality and reducing hospital costs, as has been demonstrated by previously validated large national clinical audits such as NSQuIP [36] and DCRA [10]. The Dutch experience, thanks to the inclusion of the entire colorectal surgery population, represents to date, the most meaningful one given its “real-world” nature without selection bias and with risk-adjusted outcome data.

ESCA is an initiative which follows one of the main recommendations of the European Cancer Care Organization (ECCO) — Essential Requirements for Quality Cancer Care (ERQCC). The ERQCC recommended that clinical and process outcome data should be systematically measured and collected to give high quality care to patients [37].

With the paramount of a concrete enhancement of postoperative outcomes and a reduction of costs, ESCA aims to investigate, for the first time in Italy, the impact of systematic clinical auditing and feedback in the field of colorectal cancer surgery among a large population representative of a real-world population. Key performance indicators based on evidence-based guidelines, web-based registration of clinical data made by physicians integrated with administrative data and continuous feedback on the enrolment, and risk-adjusted outcomes are the critical elements of the study, which will provide strong and reliable data to measure and improve quality of colorectal cancer surgical care.

Future challenges will be to enhance this project at regional or national level and will use our experience to set regional and national quality standards.

## Supplementary Information

Below is the link to the electronic supplementary material.

Supplementary file1 (DOC 128 KB)

## References

[1] Burgess R, Moorhead J (2020) New principles of best practice in clinical audit. Burgess, R (ed) 2nd ed. CRC Press. ISBN 9781003155393

[2] McCambridge J, Witton J, Elbourne DR (2014). Systematic review of the Hawthorne effect: new concepts are needed to study research participation effects. J Clin Epidemiol.

[3] Govednik J, McGuckin M (2011). A Multicenter trend analysis of hand hygiene event rates in USA pre- and post joint commission and World Health Organization guidelines. Am J of Infect Control.

[4] Mangione-Smith R, Elliott MN, McDonald L, McGlynn EA (2002). An observational study of antibiotic prescribing behavior and the Hawthorne effect. Health Serv Res.

[5] Meeker D, Linder JA, Fox CR, Friedberg MW, Persell SD, Goldstein NJ, Knight TK, Hay JW, Doctor JN (2016). Effect of Behavioral interventions on inappropriate antibiotic prescribing among primary care practices: a randomized clinical trial. JAMA.

[6] van de Graaf FW, Lange MM, Spakman JI, van Grevenstein WMU, Lips D, de Graaf EJR, Menon AG, Lange JF (2019). Comparison of systematic video documentation with narrative operative report in colorectal cancer surgery. JAMA Surg.

[7] Leersum NJ, Van; Snijders, H.S., Henneman, D., Kolfschoten, N.E., Gooiker, G.A., Bemelman, W.A., Dam, R.M. Van; Elferink, M.A., Karsten, T.M., Krieken, J.H.J.M. Van,  (2013). The Dutch surgical colorectal audit. Eur J Surg Oncol.

[8] Bakker IS, Snijders HS, Wouters MW, Havenga K, Tollenaar RAEM, Wiggers T, Dekker JWT (2014). High complication rate after low anterior resection for mid and high rectal cancer; results of a population-based study. Eur J Surg Oncol.

[9] Zerillo JA, Schouwenburg MG, van Bommel ACM, Stowell C, Lippa J, Bauer D, Berger AM, Boland G, Borras JM, Buss MK (2017). An international collaborative standardizing a comprehensive patient-centered outcomes measurement set for colorectal cancer. JAMA Oncol.

[10] de Neree Tot Babberich MPM, Detering R, Dekker JWT, Elferink MA, Tollenaar RAEM, Wouters MWJM, Tanis PJ, de Neree MPM, Detering R, Willem J et al (2018) Achievements in colorectal cancer care during 8 years of auditing in The Netherlands. Eur J Surg Oncol 44:1361–1370. 10.1016/j.ejso.2018.06.001 10.1016/j.ejso.2018.06.001 29937415

[11] Govaert JA, van Dijk WA, Fiocco M, Scheffer AC, Gietelink L, Wouters MWJM, Tollenaar RAEM (2016). Nationwide outcomes measurement in colorectal cancer surgery: improving quality and reducing costs. J Am Coll of Surg.

[12] Gietelink L, Wouters MWJM, Marijnen CAM, van Groningen J, van Leersum N, Beets-Tan RGH, Tollenaar RAEM, Tanis PJ (2017). Changes in nationwide use of preoperative radiotherapy for rectal cancer after revision of the national colorectal cancer guideline. Eur J Surg Oncol.

[13] AIOM, AIRTUM (2020) SIAPEC-IAP. I numeri del cancro in Italia 2020. Available on line: https://www.aiom.it/wp-content/uploads/2020/10/2020_Numeri_Cancro-operatori_web.pdf. Accessed 28 March 2022

[14] Italian Ministry of Health. Programma Nazionale Esiti. Available online: https://pne.agenas.it/main/doc/Report_PNE_2021.pdf. Accessed 28 March 2022

[15] Chan A-W, Tetzlaff JM, Altman DG, Laupacis A, Gøtzsche PC, Krleža-Jerić K, Hróbjartsson A, Mann H, Dickersin K, Berlin JA (2013). SPIRIT 2013 statement: defining standard protocol items for clinical trials. Ann Intern Med.

[16] Oken MM, Creech RH, Tormey DC, Horton J, Davis TE, McFadden ET, Carbone PP (1982). Toxicity and response criteria of the Eastern Cooperative Oncology Group. Am J Clin Oncol.

[17] Keats AS (1978). The ASA classification of physical status–a recapitulation. Anesthesiology.

[18] Owens WD, Felts JA, Spitznagel ELJ (1978). ASA physical status classifications: a study of consistency of ratings. Anesthesiology.

[19] Katz S, Ford AB, Moskowitz RW, Jackson BA, Jaffe MW (1963) Studies of illness in the aged. The index of ADL: a standardized measure of biological and psychological function. JAMA 185:914–919. 10.1001/jama.1963.03060120024016 10.1001/jama.1963.03060120024016 14044222

[20] Deschodt M, Wellens NIH, Braes T, De Vuyst A, Boonen S, Flamaing J, Moons P, Milisen K (2011). Prediction of functional decline in older hospitalized patients: a comparative multicenter study of three screening tools. Aging Clin Exp Res.

[21] Zattoni D, Montroni I, Saur NM, Garutti A, Bacchi Reggiani ML, Galetti C, Calogero P, Tonini V (2019). A simple screening tool to predict outcomes in older adults undergoing emergency general surgery. J Am Geriatr Soc.

[22] Chang C-M, Yin W-Y, Wei C-K, Wu C-C, Su Y-C, Yu C-H, Lee C-C (2016). Adjusted age-adjusted Charlson comorbidity index score as a risk measure of perioperative mortality before cancer surgery. PLoS ONE.

[23] Tian Y, Jian Z, Xu B, Liu H (2017). Age-adjusted Charlson comorbidity index score as predictor of survival of patients with digestive system cancer who have undergone surgical resection. Oncotarget.

[24] Kondrup J, Rasmussen HH, Hamberg O, Stanga Z (2003) Nutritional risk screening (NRS 2002): a new method based on an analysis of controlled clinical trials. Clin Nutr (Edinburgh, Scotland) 22:321–336. 10.1016/s0261-5614(02)00214-5 10.1016/s0261-5614(02)00214-5 12765673

[25] Clavien PA, Barkun J, de Oliveira ML, Vauthey JN, Dindo D, Schulick RD, de Santibanes E, Pekolj J, Slankamenac K, Bassi C (2009). The Clavien-Dindo classification of surgical complications: five-year experience. Ann Surg.

[26] Dindo D, Demartines N, Clavien P-A (2004). Classification of surgical complications: a new proposal with evaluation in a cohort of 6336 patients and results of a survey. Ann Surg.

[27] Slankamenac K, Graf R, Barkun J, Puhan MA, Clavien P-A (2013). The comprehensive complication index: a novel continuous scale to measure surgical morbidity. Ann Surg.

[28] Nagtegaal ID, Odze RD, Klimstra D, Paradis V, Rugge M, Schirmacher P, Washington KM, Carneiro F, Cree IA (2020). The 2019 WHO classification of tumours of the digestive system. Histopathology.

[29] Quirke P (2003). Training and quality assurance for rectal cancer: 20 years of data is enough. Lancet Oncol.

[30] Quirke P, Morris E (2007). Reporting colorectal cancer. Histopathology.

[31] Ryan R, Gibbons D, Hyland JMP, Treanor D, White A, Mulcahy HE, O’Donoghue DP, Moriarty M, Fennelly D, Sheahan K (2005). Pathological response following long-course neoadjuvant chemoradiotherapy for locally advanced rectal cancer. Histopathology.

[32] Edge SB, Compton CC (2010) The American Joint Committee on Cancer: the 7th edition of the AJCC cancer staging manual and the future of TNM. Ann Surg Oncol 17(6):1471–1474. 10.1245/s10434-010-0985-4 20180029

[33] Porter ME (2010). What is value in health care?. N Engl J Med.

[34] Hall BL, Hirbe M, Waterman B, Boslaugh S, Dunagan WC (2007). Comparison of mortality risk adjustment using a clinical data algorithm (American College of Surgeons National Surgical Quality Improvement Program) and an administrative data algorithm (Solucient) at the case level within a single institution. J Am Coll Surg.

[35] Ghaferi AA, Birkmeyer JD, Dimick JB (2009). Variation in hospital mortality associated with inpatient surgery. N Engl J Med.

[36] Ingraham AM, Richards KE, Hall BL, Ko CY (2010). Quality improvement in surgery: the American College of Surgeons National Surgical Quality Improvement Program approach. Adv Surg.

[37] Beets G, Sebag-Montefiore D, Andritsch E, Arnold D, Beishon M, Crul M, Dekker JW, Delgado Bolton R, Fléjou JF, Grisold W et al (2017) ECCO essential requirements for quality cancer care: colorectal cancer. a critical review. Crit Rev Oncol Hematol 110:81–93. 10.1016/j.critrevonc.2016.12.001 10.1016/j.critrevonc.2016.12.001 28109408

